# Supplementary material for: Co-production for the integration of migrant human capital into the decent work
Source: PLoS One. 2023 Dec 13;18(12):e0295756. doi: 10.1371/journal.pone.0295756 (PMC10718452; doi:10.1371/journal.pone.0295756)
Supplement: Attachment — Submitted filename: PLOSONE_19oct_ResponseLetter.docx [file pone.0295756.s001.docx]

**RESPONSE LETTER**

**PONE-D-23-26147**

**Co-Production for the integration of migrant human capital into the decent work**

**Dear Professor Dr. Cesar Infantile Xibile**

Editor-in-Chief of PLOS ONE

Thank you very much for considering our research paper for possible publication in the esteemed PLOS ONE. We are grateful for your oversight of this process and the reviewers' suggestions to improve our revised submission. We have made all suggested revisions and we appreciate the reviewers' suggestions and calls for more clarification regarding our submission. After careful consideration, we now resubmit this paper for your review.

This response letter addresses all the revisions as indicated in the reviewers' comments prefixed as “COMMENT:”, while our responses are prefixed with “AUTHORS’ RESPONSE:” in blue text in the main body manuscript.

We hope that this submission of our paper meets the quality standard and expectations of the PLOS ONE editors, reviewers, and readers.

Sincerely,

Valentina Gomes Haensel Schmitt

Corresponding author

**Editor comments:**

**PONE-D-23-26147**

**Co-Production for the integration of migrant human capital into the decent work**

**PLOS ONE**

Dear Dr. Gomes,

Thank you for submitting your manuscript to PLOS ONE. After careful consideration, we feel that it has merit but does not fully meet PLOS ONE’s publication criteria as it currently stands. Therefore, we invite you to submit a revised version of the manuscript that addresses the points raised during the review process.

==============================

Please submit your revised manuscript by Nov 11 2023 11:59PM. If you will need more time than this to complete your revisions, please reply to this message or contact the journal office at [plosone@plos.org](mailto:plosone@plos.org). When you're ready to submit your revision, log on to https://www.editorialmanager.com/pone/ and select the 'Submissions Needing Revision' folder to locate your manuscript file.

Please include the following items when submitting your revised manuscript:

- A rebuttal letter that responds to each point raised by the academic editor and reviewer(s). You should upload this letter as a separate file labeled 'Response to Reviewers'.
- A marked-up copy of your manuscript that highlights changes made to the original version. You should upload this as a separate file labeled 'Revised Manuscript with Track Changes'.
- An unmarked version of your revised paper without tracked changes. You should upload this as a separate file labeled 'Manuscript'.

If you would like to make changes to your financial disclosure, please include your updated statement in your cover letter. Guidelines for resubmitting your figure files are available below the reviewer comments at the end of this letter.

…
We look forward to receiving your revised manuscript.

Kind regards,

Cesar Infante Xibille, Ph.D

Academic Editor

PLOS ONE

**Journal requirements:**

When submitting your revision, we need you to address these additional requirements.

**COMMENT 1.** Please ensure that your manuscript meets PLOS ONE's style requirements, including those for file naming. The PLOS ONE style templates can be found at 
https://journals.plos.org/plosone/s/file?id=wjVg/PLOSOne_formatting_sample_main_body.pdf and 
https://journals.plos.org/plosone/s/file?id=ba62/PLOSOne_formatting_sample_title_authors_affiliations.pdf

**AUTHORS’ RESPONSE:**

The manuscript format was modified to align with PLOS ONE's style guidelines.

**COMMENT 2.** Please provide additional details regarding participant consent. In the Methods section, please ensure that you have specified (1) whether consent was informed and (2) what type you obtained (for instance, written or verbal).

**AUTHORS’ RESPONSE:**

As suggested, additional details concerning participant consent were provided in the section "3. Materials and Methods; 3.2. Procedures".

The previous version: *Also, it was clarified that participation in the interview would not imply any risk to the participants, and consent was requested both for participation and processing the information.*

The modified version (lines 191-196): *“*Also, it was explicitly stated that taking part in the interview posed no risk to the participants, and the interview content would be used only for this specific study and not be shared where else – as approved by the Ethics Committee. The study incorporated an informed consent procedure to provide participants with the necessary information to freely decide on their involvement in the research and the authorisation for using their related data. The consent was provided verbally.”

**COMMENT 3.** We note that the grant information you provided in the ‘Funding Information’ and ‘Financial Disclosure’ sections do not match.

When you resubmit, please ensure that you provide the correct grant numbers for the awards you received for your study in the ‘Funding Information’ section.

**AUTHORS’ RESPONSE:**

In the ‘Funding Information’ section, we provided the correct grant numbers for the awards we received for our study, i.e., PI.52.001.2022.

The modified version: “Funding: The authors would like to extend their appreciation to Universidad de Lima (Peru) for funding this work through the Instituto de Investigación Científica research support, under the number **PI.52.001.2022**. The funders had no role in study design, data collection and analysis, decision to publish, or preparation of the manuscript.”

**COMMENT 4.** We note that you have indicated that data from this study are available upon request. PLOS only allows data to be available upon request if there are legal or ethical restrictions on sharing data publicly. For more information on unacceptable data access restrictions, please see http://journals.plos.org/plosone/s/data-availability#loc-unacceptable-data-access-restrictions.

In your revised cover letter, please address the following prompts:

a) If there are ethical or legal restrictions on sharing a de-identified data set, please explain them in detail (e.g., data contain potentially sensitive information, data are owned by a third-party organization, etc.) and who has imposed them (e.g., an ethics committee). Please also provide contact information for a data access committee, ethics committee, or other institutional body to which data requests may be sent.

b) If there are no restrictions, please upload the minimal anonymized data set necessary to replicate your study findings as either Supporting Information files or to a stable, public repository and provide us with the relevant URLs, DOIs, or accession numbers. For a list of acceptable repositories, please see http://journals.plos.org/plosone/s/data-availability#loc-recommended-repositories.

We will update your Data Availability statement on your behalf to reflect the information you provide.

**AUTHORS’ RESPONSE:**

In adherence to the directives of the ethics committee, we have diligently apprised the interviewees of the comprehensive process of informed consent. This procedure is meticulously designed to ensure that participants are equipped with all requisite information, empowering them to make decisions regarding their involvement in the research and the authorization for the utilization of data pertaining to their person.

We have conducted essential coordination with Mr. Alexandre Almeida, the director of the Scientific Research Institute of the Universidad de Lima. Consequently, the information obtained from interviews has been anonymized to safeguard the identities of the respondents.

**Due to ethical constraints regarding data sharing, custody of the data will be entrusted to a designated non-author point of contact, namely, the Scientific Research Institute of the Universidad de Lima.** This institution will serve as the primary liaison for handling requests pertaining to data access. **The email is the following: idic@ulima.edu.pe.** Once again, thank you for your consideration

**COMMENT 5.** We note that you have stated that you will provide repository information for your data at acceptance. Should your manuscript be accepted for publication, we will hold it until you provide the relevant accession numbers or DOIs necessary to access your data. If you wish to make changes to your Data Availability statement, please describe these changes in your cover letter and we will update your Data Availability statement to reflect the information you provide.

**AUTHORS’ RESPONSE:**

After careful deliberation, we have made the decision to revise our Data Availability statement. Regrettably, we are unable to facilitate the sharing of data at this time. We appreciate your understanding in this matter.

**COMMENT 6**. Please include your full ethics statement in the ‘Methods’ section of your manuscript file. In your statement, please include the full name of the IRB or ethics committee who approved or waived your study, as well as whether or not you obtained informed written or verbal consent. If consent was waived for your study, please include this information in your statement as well.

**AUTHORS’ RESPONSE:**

In the section "3. Materials and Methods; 3.2. Procedures," information on ethics committee was provided.

The previous version: *Participants were sent a standardised message inviting to take part in the study, and informed that the selection criterion was to be a managing member of an organisation involved in the integration process of Venezuelan migrants as productive subjects in Lima’s labour market.*

The modified version (lines 180-182): *Firstly, the Ethics Committee of the Scientific Research Institute of the University of Lima, with opinion No. 027-CEI-IDIC-ULIMA-2022, has decided to approve the ethical viability of our research project. Then, participants were sent a standardised message inviting to take part in the study, and informed that the selection criterion was to be a managing member of an organisation involved in the integration process of Venezuelan migrants as productive subjects in Lima’s labour market.*

**COMMENT 7.** Please review your reference list to ensure that it is complete and correct. If you have cited papers that have been retracted, please include the rationale for doing so in the manuscript text, or remove these references and replace them with relevant current references. Any changes to the reference list should be mentioned in the rebuttal letter that accompanies your revised manuscript. If you need to cite a retracted article, indicate the article’s retracted status in the References list and also include a citation and full reference for the retraction notice.

**AUTHORS’ RESPONSE:**

We have thoroughly reviewed our reference list and can confirm that none of the papers we utilised have been retracted. However, we have taken note of your guidance and have made sure to verify the status of all cited papers.

In addition, we have incorporated the following papers to further bolster the robustness of our research (Lines 73-79):

Altonji, J. G., Card, D. (1991). "The Effects of Immigration on the Labor Market Outcomes of Less-Skilled Natives." In Immigration, Trade, and the Labor Market, edited by J. M. Abowd and R. B. Freeman. University of Chicago Press.

Card, D., Dustmann, C., Preston, I (2012): "Immigration, wages and compositional amenities." Journal of the European Economic Association, 10 (1), 78-119.

Card, D (2007). "How Immigration affects U.S. cities." No 0711, CReAM Discussion paper series, Centre for Research and Analysis of Migration (CReAM), Department of Economics, University College London.

We believe that these additions enhance the depth and scope of our research.

**Reviewers' comments:**

**Reviewer's Responses to Questions**

**Comments to the Author**

**COMMENT 1**. Is the manuscript technically sound, and do the data support the conclusions?

**Reviewer #1: Yes**

**AUTHORS’ RESPONSE:** We would like to thank you for taking the time to review thoroughly our material. We have considered all your recommendations and added them to this recent version. We hope it now meets all your expectation.

**COMMENT 2.** Has the statistical analysis been performed appropriately and rigorously?

**Reviewer #1: N/A**

**COMMENT 3**. Have the authors made all data underlying the findings in their manuscript fully available?

**Reviewer #1: Yes**

**AUTHORS’ RESPONSE:** Thank you for recognizing it.

**COMMENT 4.** Is the manuscript presented in an intelligible fashion and written in standard English?

**Reviewer #1: Yes**

**AUTHORS’ RESPONSE:** Thank you for recognizing it.

**COMMENT** 5. Review Comments to the Author

Reviewer #1: It is an interesting exploratory study on labour migration in Latin America. Overall, a sound research design, and well executed analysis. MU main problem concerns the conceptual framework on which the study is based.

Literature review presents a stunted, and arbitrarily selected angle in the presented maniuscript as it argues that “migration movements can have a positive impact on social and economic welfare of the host country, as they can rejuvenate the working population and intensify the labour force.” (Lines 91-93).

The “can” suggests that it cannot, but doesn’t have to and this raises the question of short- versus long-term consequences as well as proportionality. Being a migrant myself, I will be the last one to argue that migration does not have a positive impact on human potential but to claim that it is solely or overwhelmingly positive, would be too erroneous.

The claimed economic benefits of migrationare wildly overstated in the conceptual framework. The authors ignore or understate crowding and congestion effects as well as the importance of positional goods (amenity/status goods rationed in supply such that they gain value from others not having them). Most importantly, the manuscript obscures the downward pressures immigration puts on wages, suppressing wages rather than cutting them. The Baumol Effect means that competition for high productivity workers raises wages in occupations that do not enjoy increases in productivity. Using migration to make labour more plentiful compared to capital suppresses the Baumol effect and so suppresses wages. Hence the pandemic pause on migration led to (money) wages at the lower end of  labour markets starting to rise noticeably in the US and the UK: the Baumol Effect was no longer being suppressed. As Per Peter Turchin, it is a not a coincidence that US growth in real wages essentially stopped a few years after mass migration to the US resumed. Canada is another case showcasing this dynamic very clearly at the moment. It is hard to believe that Latin America is an outlier case. This is a dimension that should be addressed in revisions. Two pointers where to start below, but I am confident the authors will be able to find literature on the subject without any problems.

George Borjas, ‘Immigration and the American Worker: A Review of the Academic Literature,’ Center for Immigration Studies, April 2013.

David Card, Christian Dustmann and Ian Preston, ‘Immigration, Wages, and Compositional Amenities,’ Norface Migration Discussion Paper No. 2012-13, February 2012.

**AUTHORS’ RESPONSE:**

Upon careful consideration, we have chosen to replace the term “can” with “might,” thereby focusing on the potential positive implications of migration. However, to maintain a balanced perspective and acknowledge the potential challenges associated with migration, we have incorporated insights drawn from the esteemed works of researchers such as **David Card and Joseph G. Altonji.** Their studies conclude that, at the local level, immigration wields influence over a range of factors, encompassing wages, tax structures, demographic composition, and labor market dynamics. These dynamics may engender competitive elements between migrants and native residents, potentially unsettling the socio-demographic equilibrium that native inhabitants typically encounter in their daily experiences.

The previous version:

*In migration processes, the adaptation of new human capital is intended to foster positive relations between migrants and local communities with the aim of ensuring the optimal development and utilisation of its potential [35]. Integration can be understood as a ¨two-way¨ process of mutual accommodation of host communities and immigrants [36]. Socioeconomic integration of immigrants is considered one of the most important paths when seeking common prosperity, harmony, and social stability [37]. In this sense, migration movements can have a positive impact on social and economic welfare of the host country, as they can rejuvenate the working population and intensify the labour force. Therefore, it is essential that the host country provides support in terms of governance and protection of migrant workers [3].*

**The modified version (lines 71-85:**

*“In migration processes, the adaptation of new human capital is intended to foster positive relations between migrants and local communities with the aim of ensuring the optimal development and utilisation of its potential [35]. At the local level, immigration not only impacts wages and taxes but also alters the composition of the local population [36] and influences the labour market dynamics [37]. This shift may potentially disrupt the socio-demographic balance that native inhabitants experience in their day-to-day lives [36, 37] and possibly raise more concerns among individuals with lower educational attainment [36]. Moreover, the migration of individuals with lower skill levels may lead to modest competition between migrants and native inhabitants [38] . Integration can be understood as a ¨two-way¨ process of mutual accommodation of host communities and immigrants [39]. Socioeconomic integration of immigrants is considered one of the most important paths when seeking common prosperity, harmony, and social stability [40]. In this sense, migration movements might have a positive impact on social and economic welfare of the host country, as they can rejuvenate the working population and intensify the labour force. Therefore, it is essential that the host country provides support in terms of governance and protection of migrant workers [3]”*

**COMMENT 6.** PLOS authors have the option to publish the peer review history of their article ([what does this mean?](https://journals.plos.org/plosone/s/editorial-and-peer-review-process#loc-peer-review-history)). If published, this will include your full peer review and any attached files.

**Do you want your identity to be public for this peer review?**

**Reviewer #1: No**

**Response:**

Also, please, note that we have updated the author information at the cover letter:

1. Name: Mirza Marvel Cequea
2. Affiliation: Faculty of Geography and Regional Studies, University of Warsaw, Poland

Finally, we would like to extend our sincere gratitude for dedicating your time and expertise evaluating our manuscript. Your insightful comments and constructive feedback have been instrumental in enhancing the quality and rigor of our work.

We have carefully considered each of the points raised, and in response, we have made the necessary revisions to address the concerns raised. We are confident that these revisions align with your expectations and have effectively clarified pertinent aspects of the manuscript.

We greatly value the contributions of the reviewers and the editorial team in ensuring the scholarly rigor and integrity of our research. We believe that the revisions made have strengthened the manuscript and we look forward to your further guidance as we move towards the publication stage.

Thank you once again for your invaluable input and for the opportunity to contribute to the body of knowledge in our field.
